# Influence of Olfactory Epithelium on Mitral/Tufted Cell Dendritic Outgrowth

**DOI:** 10.1371/journal.pone.0003816

**Published:** 2008-11-26

**Authors:** Ha Tran, Huaiyang Chen, Andreas Walz, Jamie C. Posthumus, Qizhi Gong

**Affiliations:** 1 Department of Cell Biology and Human Anatomy, School of Medicine, University of California Davis, Davis, California, United States of America; 2 The Rockefeller University, New York, New York, United States of America; Temasek Life Sciences Laboratory, Singapore

## Abstract

Stereotypical connections between olfactory sensory neuron axons and mitral cell dendrites in the olfactory bulb establish the first synaptic relay for olfactory perception. While mechanisms of olfactory sensory axon targeting are reported, molecular regulation of mitral cell dendritic growth and refinement are unclear. During embryonic development, mitral cell dendritic distribution overlaps with olfactory sensory axon terminals in the olfactory bulb. In this study, we investigate whether olfactory sensory neurons in the olfactory epithelium influence mitral cell dendritic outgrowth *in vitro*. We report a soluble trophic activity in the olfactory epithelium conditioned medium which promotes mitral/tufted cell neurite outgrowth. While the trophic activity is present in both embryonic and postnatal olfactory epithelia, only embryonic but not postnatal mitral/tufted cells respond to this activity. We show that BMP2, 5 and 7 promote mitral/tufted cells neurite outgrowth. However, the BMP antagonist, Noggin, fails to neutralize the olfactory epithelium derived neurite growth promoting activity. We provide evidence that olfactory epithelium derived activity is a protein factor with molecular weight between 50–100 kD. We also observed that Follistatin can effectively neutralize the olfactory epithelium derived activity, suggesting that TGF-beta family proteins are involved to promote mitral/tufted dendritic elaboration.

## Introduction

The characteristic mitral cell dendritic morphology defines its function in olfactory information processing. In the mature olfactory bulb (OB), each mitral cell bears a single apical dendrite in rodents [1]–[4]. The terminals of the apical dendrite form a highly branched tuft but their distribution is restricted to a single glomerulus. This morphology ensures that each mitral cell only receives synaptic innervation from axons of the same odorant receptor expressing olfactory sensory neurons [5]–[7]. Mitral cells initiate their dendritic processes during early embryonic development [1], [3]. Elaborate dendritic trees are developed during embryonic stages while maturation of the apical dendrites happens subsequently at early postnatal stages [2], [8].

Dendritic morphology of neurons defines their functionality in the neuronal circuitry. Development of the dendritic processes is controlled by both intrinsic genetic program and interactions with the extracellular environment [9]. Neurotrophins and axon guidance proteins are shown to regulate dendritic orientation, extension and branching of cortical neurons [10]–[13]. Though molecular controls of cortical dendritic development have been investigated, the understanding of dendritic development of neurons in the olfactory system is limited.

During embryonic development, olfactory nerve terminals maintain close contact with the mitral cell dendrites [14], [15]. It has been established that olfactory sensory axons play important roles for the genesis, differentiation and survival of mitral cells in vertebrates [16]–[21]. When the olfactory placode, the primordium of the olfactory epithelium (OE), was completely or partially ablated before the formation of the olfactory bulb in *Xenopus*, the olfactory bulb either failed to form or the number of mitral cells in the OB decreased corresponding to the loss of olfactory innervation [16], [19]. Early olfactory axons penetrate into the ventricular zone of the developing OB where mitral cells genesis occurs in rodents [17]. In Pax6 loss of function mutants, OE fails to develop. Though OB like structures were observed without the presence of the OE, the characteristic protruded laminated structure did not form in Pax6 mutant mice [22]. Our understanding of the role that the OE plays in mitral/tufted cell dendritic development is limited. *In vitro* evidence indicated that the OE produced a soluble activity which could re-orient mitral cell dendrites toward them [21]. Little is known of whether the OE influences mitral cell dendrite elaboration during development or the molecular signaling pathways that are involved in this process.

In this study, we investigated whether or not the OE provides trophic support for mitral/tufted cell dendritic outgrowth and attempted to identify the molecular signals that promoted mitral/tufted cell dendritic outgrowth. Our experiments led to the discovery that OE released soluble factors which promoted embryonic mitral/tufted cell dendritic extension. We demonstrate that the response to OE derived activity differed between embryonic and postnatal mitral/tufted cells. In addition, the molecular properties of the OE derived trophic activity were characterized. We provide evidence that bone morphogenetic proteins (BMPs), members of the TGF-beta superfamily, are involved in promoting mitral/tufted cell dendritic outgrowth.

## Results

### Olfactory epithelium and olfactory bulb neuron dendrites

**Figure 1:**
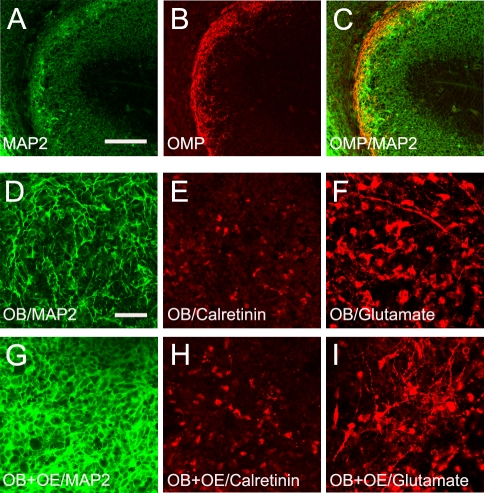
Olfactory epithelium interacts with dendrites of the olfactory bulb (OB) neurons. On a sagittal section of the E14 OB, mitral cells extend dendritic processes labeled by MAP2 immunostaining (A). Olfactory axons labeled with olfactory marker protein (OMP) expression (B) were distributed overlapping with the dendritic processes of the OB neurons (C). When olfactory bulb explants from E12 were cultured alone, dendritic processes of the OB neurons are visualized with MAP2 expression (D) and interneurons with Calretinin (E) and mitral/tufted cells with Glutamate immunostaining (F). While no increase in the interneurons (H) and glutamatergic neuron numbers was observed (I), an increase in the density of dendritic processes were observed (G) when OB explants were co-cultured in contact with the olfactory epithelium explants. Bar = 120 µm in A, 50 µm in D.

Olfactory sensory nerve axons innervate the OB preceding the genesis of mitral cells during early embryonic development [17]. Mitral cell dendritic outgrowth starts at embryonic day (E)13-E14 and continues throughout embryonic development [1]. To investigate whether mitral cell dendritic growth during embryonic development could be influenced by olfactory sensory axons, we first examined the distributions of mitral cell dendrites and the olfactory axons in the OB. At E14, mitral cells have short dendritic processes either extended perpendicular or parallel to the surface of the OB [1]. At this stage, dendritic processes of the OB neurons which were visualized by MAP2 expression showed significant overlap with the olfactory axons labeled by OMP (Fig 1A–C). This overlapping distribution supports the hypothesis that olfactory sensory axons may interact with mitral cell dendrites to influence their growth and differentiation.

To examine whether olfactory sensory axons influence the OB neuron dendritic outgrowth, we co-cultured the OB primordial explants and OE explants from E12 mouse embryos by stacking the OB explants on top of the OE explants. OB explants cultured alone were used as control. OB explants were examined by variety of markers at 5 DIV. A denser distribution of MAP2 staining was consistently observed in OB explants when co-cultured with OE compared to the control (Fig 1D–I). The dense appearance of MAP2 staining indicated that more dendritic processes were present in the OB explants co-cultured with OE. This denser dendritic process appearance could be the result of increased neuronal numbers or more elaborate dendritic processes in the OB explant. To investigate these alternatives, we used two markers to identify the number of different types of neurons in the OB explants. Mitral/tufted cells are glutamatergic neurons and are generated between E10–E14 in the OB. By counting glutamate immunopositive neurons, we observed no difference in the density of mitral/tufted cells between OB explants cultured alone when compared to that of the OB-OE co-culture (93±10 versus 90±13 cells/0.1 mm^2^, n = 3). Calretinin is expressed in a subset of the periglomerular neurons during development and in adult [23], [24]. The density of calretinin immunopositive cells in the OB explants cultured alone appeared to be similar also to that of OB explants co-cultured with OE (67±8 versus 71±5 cells/0.1 mm^2^, n = 3). These data together demonstrated that OE explants stimulated growth and elaboration of OB neuron dendrites.

### Olfactory epithelium derived activity promotes mitral cell neurite extension

OE mediated dendritic elaboration could be through a cell–cell contact mediated mechanism or via the trophic activity of diffusible factors released from OE explants. We examined whether or not the OE could release soluble trophic factor(s) by testing OE conditioned medium (OECM). To identify mitral/tufted cells in the dissociated OB culture, we utilized a transgenic mouse strain in which the endogenous neurotensin locus was replaced by neurotensin-IRES-GFP (NT-GFP) which labeled mitral and tufted cells in the OB [25]. In all dissociated OB culture experiments described in this study, only GFP expressing mitral/tufted cells were analyzed.

**Figure 2:**
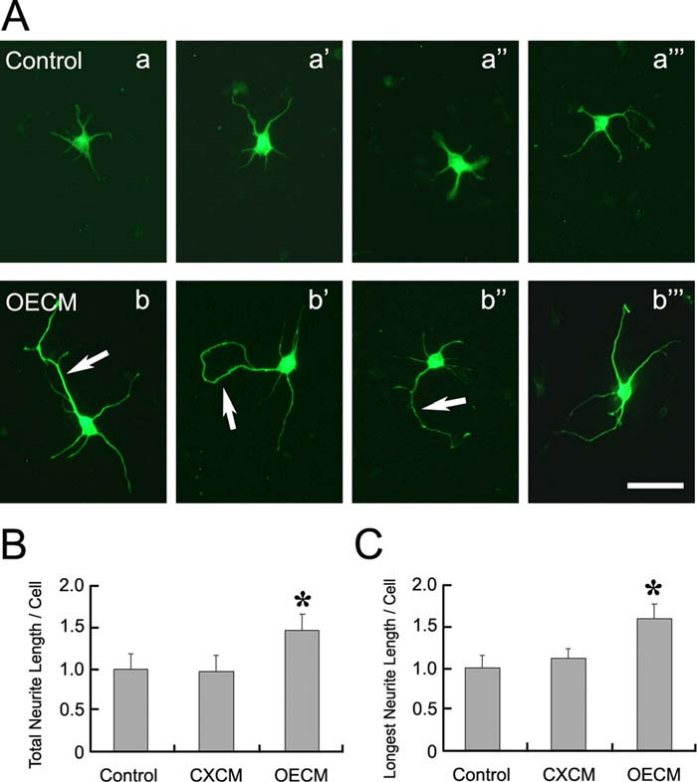
Olfactory epithelium derived activity promotes the extension of mitral/tufted cell processes. Mitral/tufted cells identified by GFP expression were dissociated from E14 olfactory bulb of NT-GFP mice (A). Mitral/tufted cells extend a few short neurite processes at 2DIV (a–a’’’) in the control. When cultured with olfactory epithelium conditioned medium (OECM), increases in neurite length were observed at 2DIV (b–b’’’) and most of them bear a single longer neurite (arrows in b–b’’). Total neurite length of each mitral/tufted cell was significantly longer (*) in OECM than the control (B) but the cortical tissue conditioned medium (CXCM) did not exhibit this effect. Longest neurite length of each mitral/tufted cell was also significantly longer (*) in OECM than the control (C). Bar = 30 µm.

To test the hypothesis that the OE derived activity is mediated by released soluble factor(s), we applied OECM, made with OE from E14 mice, to dissociated E14 OB cultures and analyzed the mitral/tufted cell dendritic morphology changes. In the control cultures, mitral/tufted cells extend multiple short processes at 2 DIV. Compared to the control culture, mitral/tufted cells in the OECM cultures exhibited more extensive neurite outgrowth at 2 DIV (Fig. 2A). The mitral/tufted cells appear to have fewer but longer neurites under the influence of OECM. Though more shorter neurites appear to extend from the control mitral/tufted cells, with the presence of OECM we observed that the total neurite length of each mitral/tufted cells was significantly longer when compared to the control, which is normalized as 1 (Control: 1±0.18, n = 160; OECM: 1.46±0.20, n = 196; Mean±SE; t-test: p<10^−5^). We observed that in the OECM cultures, mitral/tufted cells often bear one neurite that was visibly longer than other neurites from the same cell. The length of the longest neurite in OECM cultures was significantly longer that that of the controls (Control: 1±0.14, n = 160; OECM: 1.59±0.18, n = 196; t-test: p<10^−9^). To investigate whether this is a general phenomenon resulting from neuronal tissue, we tested the influence of cortical tissue conditioned medium (CXCM) on the neurite outgrowth of mitral/tufted cells. We observed that neocortical explants do not contain significant neurite promoting effect on cultured mitral cells. Neither the total neurite length (Control: 1±0.18, n = 160; CXCM: 0.97±0.19, n = 212; t-test: p>0.7) nor the length of the longest neurite (Control: 1±0.14, n = 160; CXCM: 1.10±0.12, n = 212; t-test: p>0.2) were different between CXCM and the controls. Therefore, OECM contains a soluble trophic factor that promote mitral/tufted cell neurite outgrowth.

### Developmental dynamics of the mitral/tufted cell responses to the OE derived trophic activity

**Figure 3:**
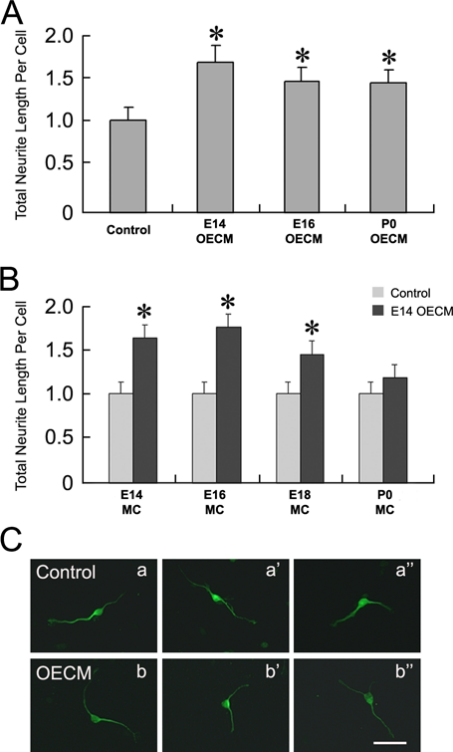
Developmental regulation of mitral/tufted cell responses to OE derived activity. (A). OE derived trophic activity was present in both embryonic (E14 and E16) and postnatal (P0) OE when tested using mitral/tufted cells from E14 OB. (B). Mitral/tufted cells (MC) from E14 to P0 OB are tested for their responsiveness to OECM (from E14 OE). Compared to neurons from the same developmental stage in the control media (normalized as 1), E14, E16 and E18 mitral/tufted cells responded to the OECM trophic activity. Total neurite length per cell was significantly increased compared to the control at each embryonic stages tested.. No significant neurite promoting response was detected when P0 mitral/tufted cells were exposed to OECM (from E14 OE). (C). Mitral/tufted cells from P0 olfactory bulb exhibit similar morphology when cultured with OECM compared to the control. Bar = 40 µm.

In the developing OB, mitral cells initiate and extend dendrites into an elaborate dendritic tree with multiple branches during embryonic development [1]. At postnatal day (P)0, however, mitral cells start to eliminate their dendritic branches into one apical dendrite bearing a dendritic tuft that extends within one glomerulus [8], [26]. This developmental difference in mitral cell dendritic growth and remodeling *in vivo* prompted the question of whether the OE derived trophic activity changes between embryonic and postnatal stages. To address this question, we evaluated whether OECM obtained by using E14, E16 and P0 OE explants, contained neurite growth promoting activity. Interestingly, when applied to E14 OB culture, OE derived trophic activity was detected in OECM from all developmental stages examined (Fig. 3A). Evaluated by the total neurite length of mitral/tufted cells, significant neurite promoting activity was detected in OECM from E14 OE (Control: 1±0.14; E14OECM 1.63±0.17; t-test: p<10^−3^), E16 OE (Control: 1±0.14; E16OECM 1.42±0.15; t-test: p<10^−3^) and P0 OE (Control: 1±0.14; P0OECM 1.40±0.14; t-test: p<3×10^−3^).

Are mitral cells from different developmental stages capable of responding to the OECM activity? In this series of experiments, mitral/tufted cells dissociated from OBs of different developmental stages were examined after being cultured with conditioned media made with E14 OE. When cultured with the presence of OECM and compared to the control mitral/tufted cells from the same developmental stage, significant increases in total neurite length were observed in mitral/tufted cells from E14 OB (Control: 1±0.13; OECM: 1.62±0.17; t-test: p<10^−3^), E16 OB (Control: 1±0.13; OECM: 1.74±0.18; t-test: p<10^−3^) and E18 OB (Control: 1±0.13; OECM: 1.44±0.16; t-test: p<0.006). Therefore, mitral/tufted cells from all embryonic stages responded to OECM derived activity by exhibiting longer neurites (Fig. 3B). Mitral/tufted cells from the P0 OB, however, fail to respond to OECM derived activity (Control: 1±0.13; OECM: 1.17±0.14; t-test: p>0.4) (Fig. 3B). The morphologies of the cultured P0 mitral/tufted cells in OECM were similar to those in the control medium (Fig. 3C).

### Identify molecular properties of the OE derived activity

**Figure 4:**
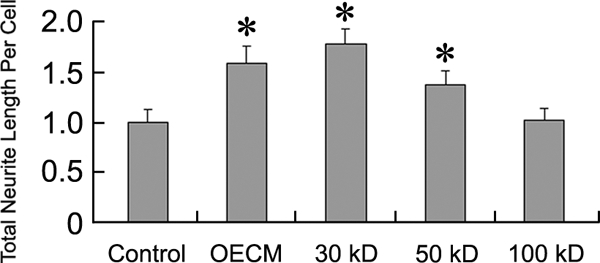
Molecular weight of the trophic activity in olfactory epithelium conditioned medium (OECM) is between 50–100 kD. OECM separated by MWCO filters was evaluated by E14 mitral/tufted cell for its neurite promoting activity. While 30 kD and 50 kD MWCO filter maintained OECM activity, the activity was lost when 100 kD filter was applied.

To identify the molecular property of this neurite growth promoting trophic activity, we first tested the stability of the activity under the treatment of trypsin and proteinase K. We observed that the OECM trophic activity is completely abolished following these treatments suggesting that the activity is likely to be exerted by protein factors (data not shown). Furthermore, OECM was fractionated using molecular weight cut-off (MWCO) filters to evaluate the molecular weight of the protein. While OECM collected by both 30 and 50 kD MWCO filters still exhibit neurite promoting activity, fractions withheld by 100 kD MWCO filter no longer contained this activity (Fig 4). This result suggests that the molecular weight of OECM derived trophic factor likely lies between the 50 and 100 kD range.

To investigate further the molecular identity of this activity, we took a candidate approach and tested a series of trophic factors which were shown to have either neurite promoting activity or were shown to be expressed in the olfactory epithelium (Table 1). Among the factors tested, neurite promoting activities were observed with NGF (100 ng/ml), BMP2 (100 ng/ml), BMP 5 (500 ng/ml) and BMP7 (100 ng/ml). Under these conditions, the total neurite lengths of the mitral cells were increased when compared to that seen in control culture, though the effect was not as strong as the OECM effect (Control: 1±0.16; NGF: 1.21±0.31; BMP2: 1.43±0.14; BMP5: 1.31±0.15; BMP7: 1.41±0.18; t-test: p<0.05 for NGF, p<0.001 for BMPs). In addition, we did not observe the morphology that was characteristic of OECM derived activity, which is the significant increase in the length of the longest neurite of each mitral/tufted cell (Fig. 2C). None of the axon guidance molecules tested, including Sema 3A, Sema 3F and Slit1, exhibited neurite promoting activity for mitral/tufted cells (Table 1). We also tested the effects of multiple factors in combination, including BMP2/7/11/Activin/GDNF, BMP2/5/7 and BMP2/NGF/NT3/BDNF. No additive effect was observed under these conditions.

**Table 1:** Trophic factors promote mitral/tufted cell dendritic outgrowth.

Factors	Concentration (ng/ml)	Bioactivity
OECM		+++
BMP2	30–100	++
BMP4	10–100	−
BMP5	10–1000	++
BMP6	30–100	−
BMP7	5–100	++
Activin A	0.01–200	−
GDF11	1–500	−
TGF-β1	0.1–200	−
NGF	10–100	+
BDNF	5–100	−
GDNF	5–100	−
NT3	10–100	−
Sema 3A		−
Sema 3F		−
Slit 1		−

Exposure to BMP2 (100 ng/ml), BMP5 (500 ng/ml) and BMP7 (50 and 100 ng/ml) showed significant bioactivity while exposure to NGF caused a slight increase in the neurite length of mitral/tufted cells.

To further test whether NGF could be responsible for the OECM activity, TrkA receptor body (TrkA/Fc), which effectively binds NGF and blocks its function [27], was applied to OECM to attempt to neutralize its neurite promoting trophic activity. No changes in the OECM activity was observed when TrkA/Fc (1 µg/ml) was pre-mixed with OECM before application (Table 2). This result in combination with the morphological difference of the mitral cells induced by NGF versus OECM suggests that NGF is not responsible for the OE derived activity. Similarly, TrkB/Fc were tested for the possible effect of BDNF and also failed to neutralize the OECM derived activity. In addition, the involvement of ionotrophic glutamatergic receptors was also excluded by using specific blockers CNQX and APV (Table 2).

**Table 2:** OECM activity is neutralized by Follistatin but not Noggin.

Neutralizing Factors	Concentration	Bioactivity
OECM		+++
TrkA-Fc	1 µg/ml	+++
TrkB-Fc	4 µg/ml	+++
APV	10 µM	+++
CNQX	10 µM	+++
Noggin	50–200 ng/ml	+++
Follistatin	100–200 ng/ml	−

**Figure 5:**
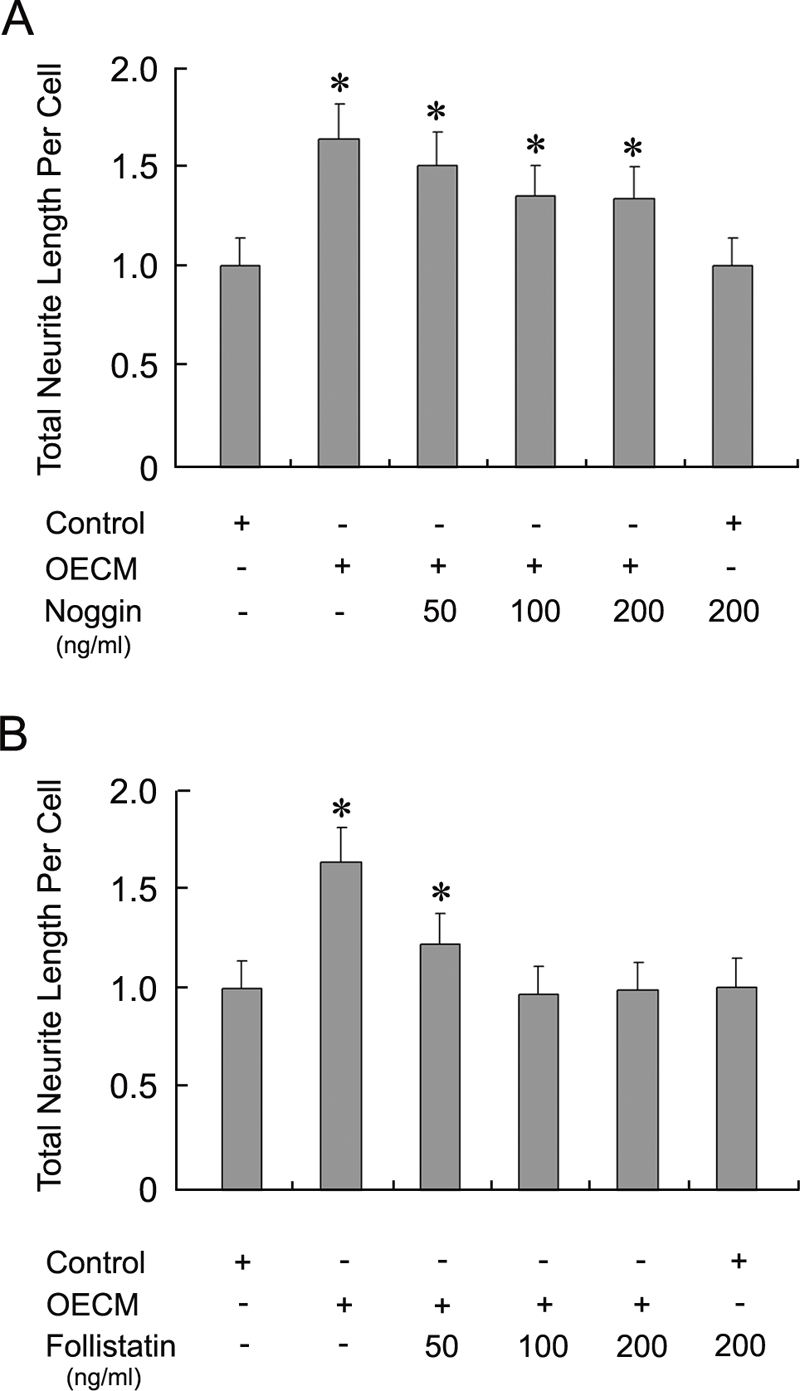
Follistatin but not Noggin neutralizes OECM activity. (A). Noggin was pre-mixed with OECM before being applied to mitral/tufted cells in culture. Compared with cultures grown with OECM, addition of Noggin (50–200 ng/ml) did not appear to reduce the trophic activity. Noggin when applied alone at 100 ng/ml, did change the growth of mitral/tufted cells. (B). Follistatin at 50 ng/ml, when pre-mixed with OECM, did not completely neutralize the OECM activity. Follistatin at 100 and 200 ng/ml completely abolished the OECM activity, while Follistatin alone did not alter the neurite length of mitral/tufted cells. * Indicated significant difference in neurite length was observed when compared to the control.

To investigate whether BMPs were responsible for the OE derived activity, we added BMP antagonists to OECM to attempt to neutralize their activity. We observed that Follistatin but not Noggin neutralized the OECM derived activity (Table 2). Neither Noggin nor Follistatin exhibited neurite growth regulating activity alone (Control: 1±0.14; Noggin100 ng/ml: 1.00±0.14; Follistatin100 ng/ml: 1.01±0.14; t-test: p>0.1). When Noggin (50–200 ng/ml) was added to OECM, OE derived activity was not reduced at 50 ng/ml (Control: 1±0.14; OECM: 1.63±0.18; Noggin50 ng/ml+OECM: 1.50±0.17; t-test: p>0.01 between OECM and Noggin+OECM) and was slightly decreased but not neutralized at 100 and 200 ng/ml (Control: 1±0.14; OECM: 1.63±0.18; Noggin100 ng/ml+OECM: 1.35±0.15; Noggin200 ng/ml+OECM: 1.33±0.15; t-test: p>0.006)(Fig. 5A). Failure to neutralize OECM activity with Noggin suggests that the identity of this activity is not due to BMP2. However, when Follistatin was added to OECM, OECM activity was significantly reduced at 50 ng/ml (Control: 1±0.14; OECM: 1.63±0.18; Follistatin50ng/ml+OECM: 1.22±0.15) and completely abolished at both 100 and 200 ng/ml (Control: 1±0.14; OECM: 1.63±0.18; Follistatin100ng/ml+OECM: 0.97±0.14; Follistatin200ng/ml+OECM: 0.99±0.14; t-test: p>0.1 between Control and Follistatin+OECM). Therefore, factors that have differential interactions with Noggin and Follistatin are likely to be responsible for the OE derived neurite promoting activity.

## Discussion

In this study we observed that OE conditioned media contained a trophic activity to promote neurite outgrowth of cultured mitral/tufted cells of the OB. We demonstrate that this trophic activity is present in both embryonic and postnatal OE. However, mitral/tufted cells from the neonatal stage unlike those from embryonic stages fail to respond to this activity. We provide evidence that the OE derived activity can be specifically blocked by Follistatin but not Noggin and that it is likely to be a protein with molecular weight between 50–100 kD. Our findings extend the observation that OE and OB interactions during embryonic stages play an important role in the differentiation of mitral cells. We propose that characterized trophic activity derived from OE promotes dendritic elaboration of mitral cells in the OB.

It has been established that olfactory sensory axons play important roles for the genesis, differentiation and survival of mitral cells in vertebrates [16]–[20], [22]. Little is known of whether or not olfactory sensory neurons play any role in regulating mitral cell dendritic development. The overlapping distribution of the olfactory axons and mitral cell dendrites during embryonic development suggest that local molecular signals may be involved to orchestrate the development of the OB. OE explant derived tropic activity was reported to reorient dendritic processes of the mitral cells in the OB explants [21]. In this study, we report a trophic activity derived from the OE which promoted the outgrowth of mitral/tufted cell neurites. Though this evidence supports the hypothesis that olfactory sensory neurons are responsible for the OE derived activity, the participation of sustentacular cells and any remaining stroma tissue in the OE explants should be considered as well. The significance of this trophic activity during olfactory system development needs to be further studied *in vivo*. In addition, we observed increased dendritic elaboration in both OB explants cultured in contact with OE explants and dissociated mitral/tufted cells cultured with OE conditioned medium. Though soluble activity is clearly present in OE condition medium, this result does not rule out the importance of contact-mediated interaction between olfactory innervation and the elaboration of mitral/tufted cell dendrites. Synaptic contacts were observed between olfactory axons and dendrites of the OB neurons at embryonic stages [28]. Several cell surface molecules mediating cell–cell adhesion or neurite growth and guidance are expressed either by the olfactory axons or OB neurons [29]–[34]. Therefore, both mechanisms could be involved in regulating the dendritic development of mitral/tufted cells.

Mitral cells in the OB exhibit different dendritic growth behavior during embryonic and postnatal stages. Throughout embryonic development, from E13 to E18, mitral cells continue to extend their dendrites into an elaborate tree [1], [3]. However, between P0–P10, dendritic trees of the mitral cells are pruned dramatically into single apical dendrite [2], [8], [26]. Evidence obtained from this study indicates that postnatal mitral/tufted cells are intrinsically different from embryonic mitral/tufted cells. Though both embryonic and postnatal OE released trophic activity, mitral/tufted cells from postnatal OB did not respond to this neurite outgrowth promoting activity. In an independent study, we obtained evidence that postnatal OB exhibited a very different transcription profile than that of the embryonic OB by genome-wide microarray screening. Many transcripts were differentially expressed between embryonic and postnatal OB (unpublished data). Several of these differentially expressed genes were specifically localized in mitral/tufted cells. In addition, odorant induced activity were shown not to be a critical player for the postnatal mitral cell dendritic pruning [2], [8]. These data together support the idea that an intrinsic genetic program is in place which regulates the elaboration and pruning of mitral/tufted cell dendrites.

Though our understanding of mitral cell dendritic development is limited, the molecular regulation of cortical neuron dendritic development has been studied [9], [10], [12], [21], [35]–[38]. Sema 3A, which is a chemorepellent for axonal growth, was shown to promote growth and orientation of apical dendrites of cortical pyramidal neurons [12]. Another repulsive molecule, Slit1, also promotes cortical neuron dendritic outgrowth and branching through Robo mediated signaling [11]. In the developing olfactory system, both Sema 3A and Slit 1 are expressed and could serve to promote mitral/tufted cell dendritic outgrowth [39]–[41]. However, no growth promoting effect was observed in cultured mitral/tufted cells when exposed to Sema 3A and Slit 1. Neurotrophins play important roles in regulating cortical neuron dendritic growth. BDNF is one of the early identified factors which promotes cortical neuron dendritic growth and branching [42]. However, BDNF did not promote dendritic growth or branching in cultured mitral/tufted cells. This difference in response to trophic factors may be simply a phenomenon resulting from culture condition differences or the age of the tissue used for the experiments. It is also possible that mitral/tufted cells have a unique genetic program to regulate their dendritic development which is different from the cortical neurons.

BMPs are members of the TGF-beta superfamily which functions pleiotropically in many developmental processes. Several BMPs are known to regulate the dendritic development of neurons both *in vitro* and *in vivo* [13], [43], [44]. For example, BMP7 promotes dendritic growth and branching for both sympathetic ganglion neurons and dissociated cortical neurons. BMP5 promotes dendritic growth for sympathetic neurons [45] while BMP2 inhibits dendritic outgrowth for postnatal cerebella neurons [46]. BMP4, BMP6 and BMP7 are expressed in the olfactory sensory neurons while BMP2 is expressed primarily in the underlining stroma tissue during embryonic development [47], [48]. Among the BMP proteins tested, we observed that BMP2, BMP5 and BMP7 promoted mitral/tufted cell dendritic extension while BMP4, BMP6 and GDF 11 did not elicit responses. BMP activities are precisely modulated by BMP antagonists which bind to BMPs with different affinity and preventing them from binding to their receptors [49]. To explore whether OE derived activity is due to functions of BMPs, we examined whether BMP antagonists could neutralized this activity. Noggin is one of the well characterized BMP antagonists that inhibits the biological functions of BMP2, BMP5 and BMP7 [49], [50]. We found that Noggin failed to neutralize the neurite growth promoting activity for mitral/tufted cells in the OECM. Biochemical evidence indicated that Noggin binds to BMP2 with high affinity but to BMP7 with low affinity. To further examine whether BMP7 could be a possible candidate for the OECM activity, we examined another BMP antagonist, Follistatin which binds to BMP7 with high affinity [51]–[53]. To our surprise, Follistatin is clearly able to neutralize the OE derived activity. This result suggests that a TGF-beta family protein, possibly BMP7, is a candidate for the OECM activity.

Is BMP7 solely responsible for the OECM activity? We reason that BMP7 can not be entirely responsible for the OE derived activity based on the following evidence: Firstly, when mitral/tufted cells were exposed to recombinant BMP7, neurite growth promoting activity was detected but was weaker and did not show the characteristic activity of OECM. Secondly, Follistatin is also an antagonist of other TGF-beta family proteins in addition to BMP7. Follistatin not only has high affinity to Activin but also binds to the heparin sulfate side chains of other proteoglycans [49], [54]. Therefore, neutralization by Follistatin may be the result of its binding to other protein factors in the OECM. Thirdly, we analyzed the protein components of OECM by liquid chromatography with tandem mass spectrometry detection method (LC-MS/MS). Preliminary data were obtained. Among 208 proteins detected by LC-MS/MS, we did not detect BMP7 in OECM (data not shown). It is possible that BMP7 is present at a level below the detection of LC-MS/MS. However, taken together these data suggest that other protein factors may be involved through either cooperation with BMP7 or independent action to promote mitral/tufted cell neurite outgrowth. Future studies will be directed to investigate protein factors identified in the OECM by LC-MS/MS for their ability to promote mitral/tufted neurite outgrowth.

## Methods

### Animals

C57/BL6 mice were purchased from Charles River Laboratories (Wilmington, MA). Neurotensin-GFP (NT-GFP) knock-in mice were used for easy identification of mitral/tufted cells in all dissociated OB culture experiments [25]. E0 was defined by the day when the copulation plug was detected in the morning. The day of birth was defined as P0. All experimental procedures were conducted according to institutional and NIH guidelines and were approved by the Institution Animal Care and Use Committee.

### Explant culture

OB and OE explants from E12 mouse embryos were dissected in ice cold calcium and magnesium free Hank's balanced salt solution (CMF-HBSS). Underlining mesenchymal tissue was carefully dissected away from the OE. OE explants were placed on the collagen coated Millicell-CM (Millipore, Billerica, MA) insert with the mucosal side facing the membrane. After allowing OE explant to attach for 2 hours, OB explants were placed on top of the OE explants with the ventricular side facing away from the OE. In control cultures, OB explants were directly placed on the Millicell-CM inserts with the ventricular side facing away from the membrane. Cultures were maintained in DMEM (Invitrogen, Carlsbad, CA), 2 mM glutamine and 10% fetal bovine serum (Invitrogen) for 5 days *in vitro* (DIV).

### Olfactory epithelium conditioned medium (OECM)

OE from E14-P0 were dissected and treated with 2 mg/ml Dispase for 30 min. After digestion, the neural epithelia were carefully separated from the underlying stroma and were placed in the Millicell-CM inserts. For cortical tissue conditioned medium (CXCM), neocortical explants from E14 were used. Cultures were maintained in phenol red free Neurobasal medium with 2 mM glutamine, N-2 supplement, and gentamycin (Invitrogen). Conditioned medium was collected after 2 DIV. Subsequently, OE and cortical tissue explants were dissociated with 0.25% trypsin and the cell numbers were counted to normalize for the strength of conditioned media according to the number of cells in the explants. Equivalent amount of the conditioned medium was added to the OB culture.

Fractionation of OECM was done using centrifugal filtration devices. After a brief centrifugation at 2000 g, the supernatant of the OECM was further separated according to its molecular weight. OECM components with their sizes larger than 30, 50 and 100 kD were concentrated and collected respectively using Centricon Ym-30, 50 and 100 (Millipore, Billerica, MA). Collected OECM fractions were subsequently tested for their neurite promoting activity in dissociated olfactory bulb culture.

### Dissociated olfactory bulb culture

OBs from E14-P0 mouse embryos or pups of NT-GFP strain were dissected in HBSS and carefully dissociated with 0.25% trypsin. 1.5×10^5^ cells were plated on each glass coverslip pre-coated with poly-D-Lysine and laminin. Dissociated OB cultures were maintained in Neurobasal medium with glutamine, N-2 supplement, B-27 and gentamycin for 2DIV. Candidate factors were added 2 hours after plating in all experiments. At 2DIV, OB cultures were fixed with 4% paraformaldehyde for 15 min and followed with standard immunocytochemistry for GFP expression to evaluate mitral/tufted cell morphology.

Candidate factors tested are the following: CNQX, D(-)-2-Amino-5-phosphonopentanoic acid (APV), Brain-derived neurotrophic factor (BDNF), Nerve growth factor (NGF), Glial cell line-derived neurotrophic factor (GDNF), NT3, TrkB/Fc receptor body were from Sigma (St. Louis, MO). Recombinant BMP2, 4, 5, 6, 7, GDF11, Activin-A, TrkA/Fc, Noggin and Follistatin were from R&D systems (Minneapolis, MN). TGF-β1 was from Peprotech (Rocky Hill, NJ). Expression plasmid for Slit1 was a gift of Dr. D. M. Ornitz.

### Immunocytochemistry

OB explants were fixed with 4% paraformaldehyde for 30 min at room temperature. Together with the underlining Millicell membrane, the explants were processes as a floating blocking for immunostaining. For Calretinin immunostaining, explants were pretreated with 0.01% pronase for 30 min before proceeding with the normal horse serum blocking. Explants were washed with phosphate buffered saline with 0.03% Triton-100 (PBS+TX), blocked with 5% normal horse serum for 1 hour at room temperature; then incubated with primary antibodies overnight at 4°C. Following washes with PBS+TX, explants were incubated with Cy2 or Cy3 conjugated secondary antibodies overnight at 4°C. Subsequent to extensive rinsing in PBS, explants were carefully separated from the Millicell membrane, mounted on slides using Fluoromount G (Fisher Scientific, Pittsburgh, PA) and examined by Zeiss LSM510 confocal microscopy. Primary antibodies and their working dilutions are: mouse monoclonal anti-MAP2 (Sigma) at 1∶1000; rabbit polyclonal anti-Glutamate (Sigma) at 1∶1000; and rabbit anti-Calretinin (Chemicon, Temecula, CA) at 1∶1000. Cy2 conjugated donkey anti-mouse and Cy3 conjugated donkey anti-rabbit antibody were used at the dilution of 1∶200 and 1∶300 respectively (Jackson Immuno, West Grove, PA).

Fronzen sagittal OB sections and dissociated OB cultures were processed for immunocytochemistry according to standard protocol. Primary antibodies used in these experiments were rabbit anti-GFP (Molecular Probes)at 1∶3000 and goat anti-olfactory marker protein (OMP) (kind gift of Dr. F. L. Margolis) at 1∶2000.

### Data Analysis

Random fields of the dissociated OB cultures were photographed. Neurite length of GFP positive cells were measured using Scion image software. All neurites were measured for each mitral/tufted cell selected. Total neurite length per cell was obtained by addition all neurite length from each cell. To minimize inter-experimental differences, the neurite length of each control experiment was normalized as 1 and compared with the experimental groups which were done in parallel. All data were pooled from at least three independent experiments and neurite length from the mitral/tufted cell was presented as Mean±Standard Error (SE). Statistical analyses were performed using student t-test.
